# Patient Complaint Cases in Primary Health Care: What Are the Characteristics of General Practitioners Involved?

**DOI:** 10.1155/2013/807204

**Published:** 2013-08-21

**Authors:** Søren Birkeland, Rene dePont Christensen, Niels Damsbo, Jakob Kragstrup

**Affiliations:** ^1^Research Unit for General Practice, Institute of Public Health, University of Southern Denmark, Denmark; ^2^Department and Research Unit of General Practice, Institute of Public Health, University of Copenhagen, Denmark

## Abstract

*Background.* Limited knowledge exists about factors increasing the risk of general practitioners becoming involved in a complaint case or getting disciplined in connection with a complaint case. *Aim.* The present study aimed to identify the general practitioner and practice characteristics associated with complaint cases and discipline. *Methods.* Information on general practitioners involved in complaint case decisions during one year (2007) was linked to Danish National register data on all general practitioners (*n* = 3,765). Logistic regression was used for statistical analysis. *Results.* With regard to complaints concerning daytime services (*n* = 265), the professional seniority of the general practitioner was positively associated with the odds of receiving a complaint decision (OR = 1.44 per 20 years of seniority; CI 95%, 1.04–1.98). Likewise, having more consultations per day was associated with increased odds (OR = 1.29 per 10 extra consultations per day; CI 95%, 1.07–1.54). No statistically significant association could be demonstrated between being disciplined and general practitioner or practice characteristics. *Conclusion.* The possible relationship between professional seniority, rate of consultations, and complaint cases merits further studies to clarify the impact of professional seniority and workload on professional performance and to furthermore consider the role of factors such as job content and communication styles.

## 1. Introduction

Patient complaints about health care may be regarded as indicators of patient dissatisfaction and shortcomings in health care provision and may therefore be used to recognize areas in the health care system, which warrant attention [1]. Most health care systems give patients the opportunity to file complaints, and in a number of countries, special patient complaint boards have been organized [2–5]. The systems may differ, and in some countries separate systems have been developed for financial compensation of patients. It is, however, a common feature that the complaint boards have the right to impose disciplinary sanctions (most often critique) on the health staff providing the service subject of complaint. 

Recent research suggests that the number of complaints about the physician-patient relationship is increasing [1]. A substantial amount of complaints concern general practice; hence, in Denmark, more than one fifth of complaints concerned general practice in 2007 [6]. In general practice the physician-patient relationship is of particular importance. Limited knowledge exists, however, about the predictors of complaints in general practice and, for example, what are the characteristic of general practitioners (GPs) involved in complaint cases. We found no studies concerning GP or practice predictors of disciplinary sanctions specifically, but studies involving all medical specialties [7–9] have shown an increased risk of receiving a disciplinary sanction among male doctors. Two studies also suggested an increased risk of sanctions among senior doctors [7, 8], but one contradicting study suggested a decreased risk [9]. 

The aim of the present study based on Danish registers was to analyze the characteristics of GPs receiving a complaint or being disciplined by the complaints board. 

## 2. Materials and Methods

A register-based survey covering the year 2007 was designed in order to compare those GPs who received a decision from the complaints board with all other Danish GPs. In Denmark, GPs generally perform according to contract with the public funding authorities [10]. Thus, the sample was defined as GPs, who had a contract with the Danish health care system on 2 January 2006, and identified by means of the GP Register of the Danish National Board of Health [11]. GPs who received a complaint were identified manually by reading the files of all GP-related patient complaints finalized by the Danish Patient Complaints Board in 2007. 

Denmark has a comprehensive health care system, which is funded through tax contributions. Danish citizens are entitled to free medical care and can choose their own GP within the municipality. The GP serves the role as a “family doctor” (or “family medical practitioner”), and most citizens choose one of the GPs listed on the municipality's list. It is possible to change GP according to preferences. In 2006, more than 99% of the Danish population was listed with one of 3,765 GPs working in approximately 2,200 local single-handed or partnership practices. GPs provide basic health care including examinations, routine treatment, and health care advice and also act as gatekeepers in relation to the secondary health care system (practicing specialists and hospitals). GPs are responsible for the care of all registered patients at all hours. The GPs within a region collaborate about the out-of-hours services, where the GPs on call answer emergency calls and make home visits. 

If dissatisfied with health professionals (e.g., GPs), patients and their relatives can decide to file a written complaint. There is no fee for filing a complaint. A complaint board (until 2010 designated the “Sundhedsvæsenets Patientklagenævn,” now “Sundhedsvæsenets Disciplinærnævn,” under “Patientombuddet”) handles complaints about professionals who are authorized by the National Board of Health including GPs. At the initial stage, the board's secretariat clarifies the issues of the complaint. In this connection, the involved health professionals are obliged to provide any information to be used for the clarification of the case. Subsequently, the case may be evaluated by one of the board's consultants and a proposal is produced for the decision which is finally made by the board. The board is chaired by a judge and in addition comprises two health professionals and two laymen representing the health care users and the hospital owners, respectively. The board may impose a “discipline.” The modes of discipline which have been most commonly used are criticizing or disputing professional conduct. The latter measures imply that the health professional(s) involved, the complainant, and other relevant partakers receive the board's disapprobation of the health professional by letter. Intermittently, a discipline with injunction may be imposed (and the health professional's name may be publicly announced). Additionally, decisions finally may result in withdrawal of the authorisation to practice or bringing the GP for the prosecuting authority. In Denmark, the patient complaint system is separate from the compensation system. 

Complaint cases concerning treatment in general practice and completed by the complaints board in 2007 were retrieved from the files of the board and reviewed. The identity of all GPs receiving a complaint was noted together with the board's decision (discipline or no discipline). 

Information about the characteristics of GPs and practices (2006) was obtained from the GP Register of the Danish National Board of Health [11], the Danish Health Information database [12] and the Danish Ministry of Welfare database [13], and included GP and practice identification codes, GP gender, professional seniority (years from graduation), and practice size in terms of number of GPs working together in the practice. The practice number of consultations per three months and practice size was used to calculate the GP output per day. The general practice location was described according to three municipality level variables: socioeconomic index, senior citizen proportion, and level of urbanization. The socioeconomic index variable is an index referring to relative municipal expenditures and is based upon a number of socioeconomic parameters (e.g., proportions of unemployed citizens aged 25–59, psychiatric patients, low-income groups) [13]. This measure has been commonly used as standard measure for the state and municipalities in Denmark [14]. The senior citizen proportion variable is defined as the percentage of the municipality population aged +65 [13]. Finally, the level of urbanization variable refers to the percentage of the number of inhabitants in towns with at least 200 inhabitants of the total number of inhabitants in the municipality as of 1 January [13]. 

For the main analysis, we only included complaint cases involving daytime services, because no national information about GPs providing out-of-hours services is available. Hence, it was not possible to decide what fraction of providers that was at risk of receiving an out-of-hours patient complaint. Data were analyzed by means of logistic regression using STATA. The dependent variable in the model distinguished those who received a complaints decision or a decision on discipline from those who did not. Odds ratios (ORs) of receiving a complaints decision or being disciplined with regard to the characteristics (independent variables) mentioned previously were estimated. A probability level of *P* < 0.05 was considered statistically significant.

## 3. Results

In total, the sample comprised 3,765 Danish GPs (65% male) included in the Danish National Board of Health Register. The average professional seniority of participating GPs was 25.5 years (range 2.8–56 years). 

The board completed the handling of 265 complaints against GPs in 2007 concerning the daytime services. The associations between receiving a complaints case concerning daytime services and GP and practice characteristics are shown in Table 1.

### 3.1. Table 1

For daytime services, high professional seniority of the GP was significantly associated with increased odds of being involved in a complaint case. An increase in professional seniority of 20 years corresponded to a 44% increase in odds of receiving a complaint within one year. Also, GPs who had higher GP output per day had higher odds of being involved in a complaint; thus, an increase of 10 consultations per day resulted in a 29% increase of odds. No statistically significant associations were found for the other characteristics: gender, practice size, socioeconomic index, senior citizen proportion, or level of urbanization.

The association between disciplinary action and GP and practice characteristics is shown in Table 2.

### 3.2. Table 2

Among the 265 GPs who received a complaint case decision concerning daytime services, 71 received a discipline from the board (53 conclusions on criticism; professional conduct disputed in another 18 cases). None of the characteristics showed statistically significant associations with odds of receiving a discipline. 

An extra-analysis including complaints about the out-of-hours service (*n* = 154, table *not* included) showed that complaints apparently were more frequent for male GPs, but the relative amount of out-of-hours work performed by male and female GPs was unknown (see what is mentioned earlier). 

## 4. Discussion

In this survey, we observed two GP characteristics statistically significantly associated with being involved in a complaint case: professional seniority and GP output per day. Only when including the out-of-hours complaint cases without any knowledge about which GPs were at risk of such a complaint, male GPs appeared to have increased odds. No statistically significant association could be demonstrated between, for example, environment characteristics (including population density) and involvement in complaint cases. Likewise, no statistically significant association could be demonstrated between being disciplined and GP and practice characteristics.

The analysis was only taking into account complaint cases completed by the complaints board. The approximately one fifth of the total number of patient complaints rejected by the complaints board has not been taken into consideration. Typical reasons for complaint rejection are complaints about the level of service (e.g., waiting time) or claims for compensation without a complaint about professional conduct. Additionally, only register-based data on practice location were taken into consideration. The concrete position of the practice concerned, patient ages, and socioeconomic information have not been dealt with. Likewise, more complex issues with regard to, for example, differences in patient list compositions have not been taken into consideration.

The study represents all complaint cases concerning GPs in Denmark completed during one year and is based on reliable register data and case files. Anyhow, the multiple logistic regression analysis revealed rather broad confidence intervals and the possibility of deflation of the statistical power. Correspondingly, the sample size precludes further statistical analysis considering, for example, the role of effect modifiers and interaction among variables. Register data from 2006 were used in order to best reflect the situation when a health care event resulted in a complaint case. Thus, we expected lag times with regard to both filing the complaint and complaints board case management. However, some of the events might actually have taken place with an unfortunate time relation to the register data. As mentioned previously, subsequent to the study period, a specialized complaints board continuously considers complaints about individual GPs. The system has, however, been supplemented with additional options for those who wish to file a complaint concerning the course of health care provision (no litigation against named GPs) and the possibility of a “dialogue” with the regional municipality in order to clarify the course of concrete health care. 

Workload has formerly been measured in terms of an average number of consultations per time unit [15, 16]. In case of single-handed practices, personal number of consultations equals practice number of consultations. Although in the present study potential shortcomings may arise as consultations in partnership practices were equally distributed across the partners regardless of what was the actual partner involvement in the practice and no specific consideration is given to, for example, which GPs are working full time and which GPs are working part time. It may be argued that strictly speaking the analysis is measuring the impact of working in practices with high numbers of consultations.

Only limited literature concerning risk factors of receiving complaint cases in general practice is available. Cunningham et al. [17] carried out a cross-sectional survey among 1,200 medical doctors in New Zealand. A total of 49% (598) completed the questionnaire and were included. The study comprised a broad range of medical specialties; only 215 participants were GPs and 93 had never received a complaint. Among the broad group of medical doctors, those who were more likely to receive a complaint were GPs, male doctors, higher professional seniority doctors, and those with higher postgraduate qualifications. The authors put forward the possible explanation that it is the more experienced doctors who carry the burden of responsibility for patient care. The site of practice was of no importance.

The significance of high GP output per day suggested in the present study is supported by the findings of Nash et al. [18]. They performed a self-report study among 1,239 Australian GPs. There were 566 respondents (45.7%), and in this group the authors demonstrated that male medical doctors and doctors working more hours per week were predominant among those having had a medicolegal matter.

The present findings suggesting that complaints cases concerning male GPs are particularly preponderant only when including the out-of-hours services confront the common notion that male medical doctors are generally at a higher risk of receiving patient complaints. Unfortunately, we do not know what proportion among the GPs listed with the National Board of Health participated in the out-of-hours services and thus were at risk of being involved in an out-of-hours complaint decision; no Danish national statistics about GPs participating in the out-of-hours services are available. Consequently, it cannot be ruled out if any gender preponderance is due to the gender in itself or results from a skewed job profile, for example, if male GPs generally perform the scope of work associated with a higher risk of complaints. The out-of-hours service involves a high-risk job with regard to patient complaint cases. In the present study, more than one third of all complaint cases pertained to the out-of-hours services even though no more than approximately one tenth of general practice care pertained to out-of-hours services in 2006 [19] which suggests that, from the point of view of patient complaints, the out-of-hours services merit particular awareness.

Also, the fact that no statistically significant association could be demonstrated between being disciplined in connection with a complaint case and GP and practice characteristics confronts previous research findings [7–9].

## 5. Conclusions

It has been mentioned previously that traditional disciplinary board (and patient compensation) structures are currently complemented by new options of dialogue and modes of reconciliation in addition to possibilities to complain about courses of health care (rather than about named GPs), no-blame registration of unintended adverse events, and so forth. Anyhow, the disciplinary (complaint) system has been continuously retained as it is, for example, believed to work as a further incentive for health professionals to “do their best.” Hence, it may serve an individualised preventive function for those complained about (and especially those who have been disciplined). Accordingly, the “Patientombuddet” institution recently maintained that “*there is no doubt that those health professionals receiving a criticism for health care provision will become more attentive in future similar situations*” [20]. Likewise, more generally, it may possibly serve as a preventive measure for the broad group of health professionals. 

Additionally, as mentioned in the introduction, patient complaints and disciplinary decisions may indicate areas in health care provision that should be attended to. It appears that higher professional seniority and having more output per day increase the GP's odds of being involved in complaint cases. Nevertheless, the study suggests that the mechanisms associated with complaint cases may be complex. In this regard, a wide palette of factors, such as GP performance and communication styles, differences in job content, patient- and illness-related factors may come into play. Studies are needed to consider the role of these factors and to further clarify what lies behind the increased odds of involvement in complaint cases among GPs of higher professional seniority and GPs with higher workload. 

## Figures and Tables

**Table 1:** Receipt of a complaint case and association with general practitioner and practice characteristics^1^.

	OR	*P*	95% Confidence interval	
General practitioner characteristics				
Gender				
Female	1			
Male	0.97	0.82	0.73	1.29
Professional seniority^2^	1.44	0.03	1.04	1.98
GP output per day^3^	1.29	0.01	1.07	1.54

Practice and practice environment characteristics				
Practice size	0.99	0.86	0.91	1.08
Socioeconomic index	1.61	0.16	0.83	3.13
Senior citizen proportion	0.99	0.76	0.93	1.05
Level of urbanization	0.99	0.09	0.97	1.00

^1^Total number of Danish general practitioners, *n* = 3,765; number of complaint cases, *n* = 265.

^2^Per 20 additional years of professional seniority since graduation.

^3^Per 10 additional consultations per day. Average number of basic consultations per day per GP was 22.3 cons/(day∗GP).

**Table 2:** Disciplinary action and association with general practitioner and practice characteristics*.

	OR	*P*	95% Confidence interval	
General practitioner characteristics				
Gender				
Female	1			
Male	0.97	0.91	0.56	1.67
Professional seniority	1.85	0.06	0.98	3.49
GP output per day	1.31	0.11	0.94	1.82

Practice and practice environment characteristics				
Practice size	0.95	0.58	0.81	1.13
Socioeconomic index	0.71	0.59	0.21	2.44
Senior citizen proportion	1.00	0.95	0.90	1.12
Level of urbanization	1.01	0.65	0.98	1.03

*Total number of Danish general practitioners, *n* = 3,765; number of decisions on discipline, *n* = 71.
